# It is time to ban flavor capsule cigarettes

**DOI:** 10.18332/tpc/150334

**Published:** 2022-06-09

**Authors:** Yvette van der Eijk

**Affiliations:** 1 Saw Swee Hock School of Public Health, National University of Singapore, Singapore

**Keywords:** flavored cigarettes, industry documents, marketing, patents, tobacco industry, youth smoking

Flavor capsule cigarettes, which contain a crushable capsule in the filter, are rapidly gaining popularity especially among young people. Internal tobacco industry documents and patents reveal that this popularity is no accident as the industry’s primary target for early ‘Camel Crush’ capsule cigarettes was young millennials. Tobacco companies have patented a huge variety of flavor capsule designs, far beyond what is currently on the market, suggesting that they may launch more novelties in the near future. To protect young people from tobacco, it is essential to ban flavor capsule cigarettes and pre-empt the launch of other novelty cigarette designs.

## Flavor capsule cigarettes are gaining in popularity

Flavor capsule cigarettes, which contain a crushable flavor capsule in the filter, are the fastest growing segment of the combustible tobacco market with a market share of over 30% in some countries^1^. Their appeal among young people, as shown in studies from various countries^2-8^, is especially concerning as tobacco companies have a well-established history of targeting youth with flavored cigarettes^9,10^.

## Flavor capsule cigarettes target young people

In a 2021 study^11^, we analyzed 179 internal tobacco industry documents and 65 unique patents to understand, from the tobacco industry’s perspective, industry strategies related to flavor capsule cigarettes. Industry documents revealed that their popularity among young people is no accident. In the 2000s, when R.J. Reynolds (RJR) was preparing to launch ‘Camel Crush’, RJR had carefully studied its target market: young millennials in their teens and early 20s at the time. RJR described millennials as a culturally diverse, urbanized and ‘tech-savvy’ generation seeking novelty, individuality and a sense of control; much like today’s generation Z. Hence, RJR expected the novelty and personalization features of ‘Camel Crush’ to appeal to millennials.

In its launch of ‘Camel Crush’, RJR drew strongly on these marketing themes with youthful, ‘clubby’ imagery, emphasis on the ability to control and personalize flavor, and a ‘seed and spread’ campaign to encourage word-of-mouth marketing. This strategy was, according to RJR’s market reports, a resounding success^12,13^. Similar marketing themes persist with today’s flavor capsule cigarettes^14^, which suggests that tobacco companies continue to target young people in a similar way.

## Tobacco industry likely to market more novelty designs

Although most flavor capsule cigarettes on the market contain one or two large crushable capsules, tobacco companies have patented cigarettes with flavored granules, threads, microcapsule coatings, heat-triggered capsules, and filters that can be pulled, twisted, crushed, or covered to alter the smoke intensity, nicotine delivery, and flavor. The potential flavors and additives listed in patents are extensive, effectively including any kind of compound that can alter taste, mouthfeel, moistness, temperature sensation, smell, aroma, nicotine delivery, or other sensory characteristic^11^. Some of these novelty designs are already on markets. ‘L&M U-Spin’ has a filter that, when twisted, alters the nicotine delivery, while ‘LD Frozen’ contains flavored granules. Many other designs have been patented but not yet marketed, suggesting that tobacco companies may launch them in the near future.

Tobacco companies have also developed loose flavor capsule units, designed to be inserted into cigarette filters or packs, to flavor cigarettes. Most of these were patented recently, in the 2000s and 2010s. Some of these designs are already on the market, especially in the United Kingdom, European Union and Canada, where menthol and other flavored cigarettes are banned. Philip Morris patented flavor capsule-containing filter ends and cartridges designed to be inserted into a cigarette, while British American Tobacco patented flavor capsule units designed to be inserted into a recessed filter, already featured in some of its ‘Dunhill’ variants. RJR patented a ‘flavor additive accessory’ which, when inserted into a cigarette stick, transforms it into a flavor capsule cigarette.

## It is time to ban flavor capsule cigarettes

Tobacco companies face increasing restrictions on tobacco advertising, promotions, sponsorships and packaging. As they run out of ways to target youth, they appear to be intensifying their marketing efforts on the cigarette stick itself with colorful cigarette sticks, new flavors, and novelty product features^15^. They also appear to be using product novelties to undermine the impact of tobacco taxes^5^, plain packaging^14,16^, and tobacco flavor bans^17^. To protect young people from tobacco, it is essential to ban flavor capsule cigarettes. Given the broad and ever increasing variety of product designs, which includes accessories that can be sold separately from cigarettes, regulations should be broadly worded to include not only flavor capsule designs currently on the market, but also related designs and products that tobacco companies may market in the future.

## Data Availability

Data sharing is not applicable to this article as no new data were created.
